# Coronary Artery Fistula Ligation Using Selective Coronary Artery Angiography

**DOI:** 10.7759/cureus.8757

**Published:** 2020-06-22

**Authors:** Layla Shanah, Orazio Amabile, Sohaip Kabashneh

**Affiliations:** 1 Internal Medicine, Wayne State University/Detroit Medical Center, Detroit, USA; 2 Cardiothoracic Surgery, Banner University Medical Center Phoenix, Phoenix, USA

**Keywords:** coronary artery fistula, coronary artery angiogram

## Abstract

A 55-year-old male presented with worsening shortness of breath and was found to have multiple coronary artery fistulas on coronary angiogram with coronary steal. He subsequently underwent successful ligation of three of the fistulas using intraoperative coronary angiography in the hybrid suite to assist with the identification and confirmation of closure. There are currently no formal recommendations for the use of intraoperative imaging in such cases, but the results of our case contribute to the sparse body of literature supporting the utilization of intraoperative angiography in ligating multiple coronary artery fistulas.

## Introduction

Coronary artery fistulas (CAFs) are rare with an incidence of 0.002% among the general population, occurring in males and females equally [1]. Although they can be acquired secondary to trauma or iatrogenic injury, the majority are congenital and tend to exist as a single fistula. Surgical ligation is often reserved for those patients with significant left-to-right shunting and can be complicated by tortuous anatomy and multiple sites of origin and insertion [2,3]. We report a case of multiple CAFs successfully ligated using intraoperative coronary angiography in the hybrid suite as a guide to identify and ensure closure of each fistula.

## Case presentation

**Figure 1:**
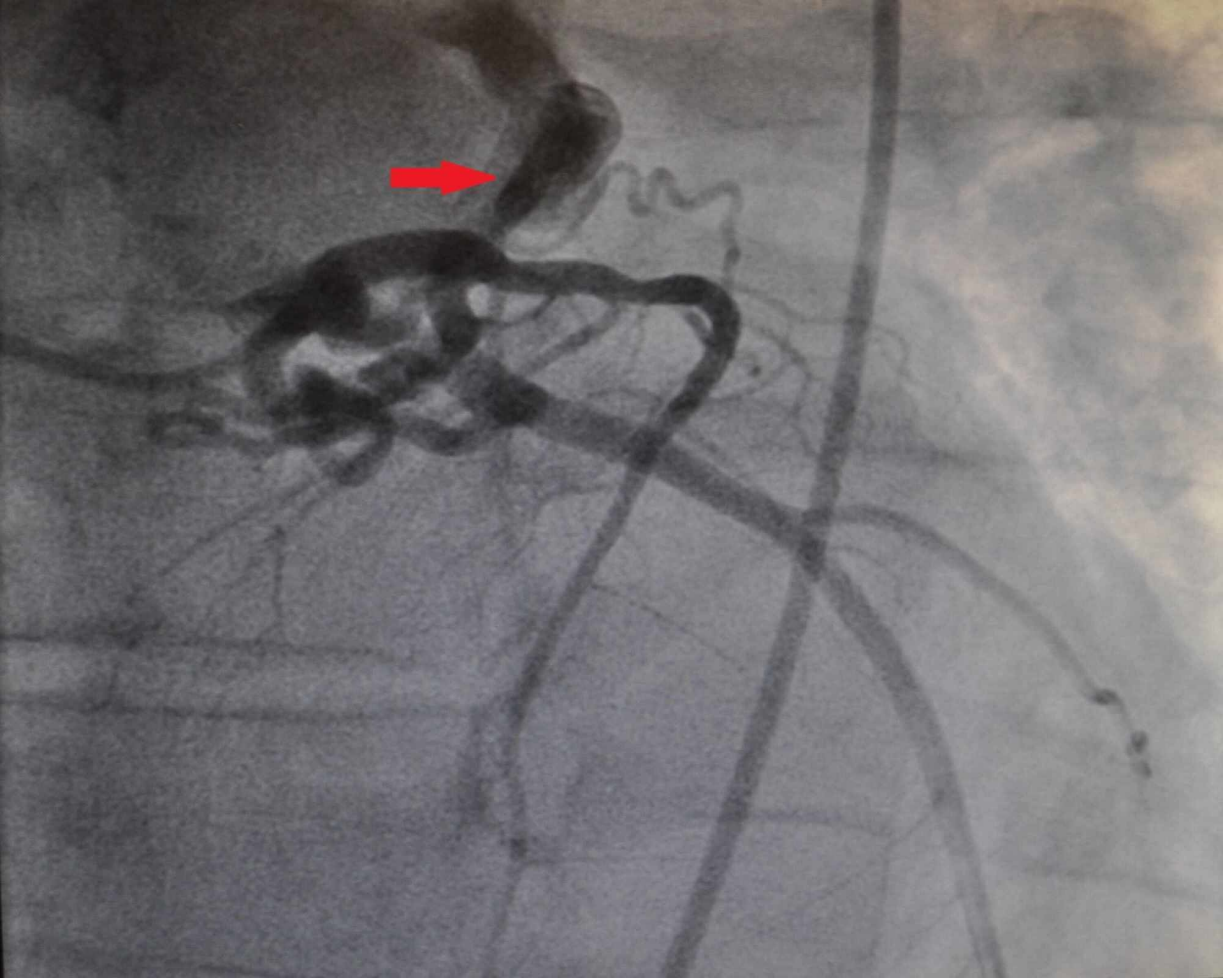
Left Coronary System to Pulmonary Artery Fistula (Red Arrow)

**Figure 2:**
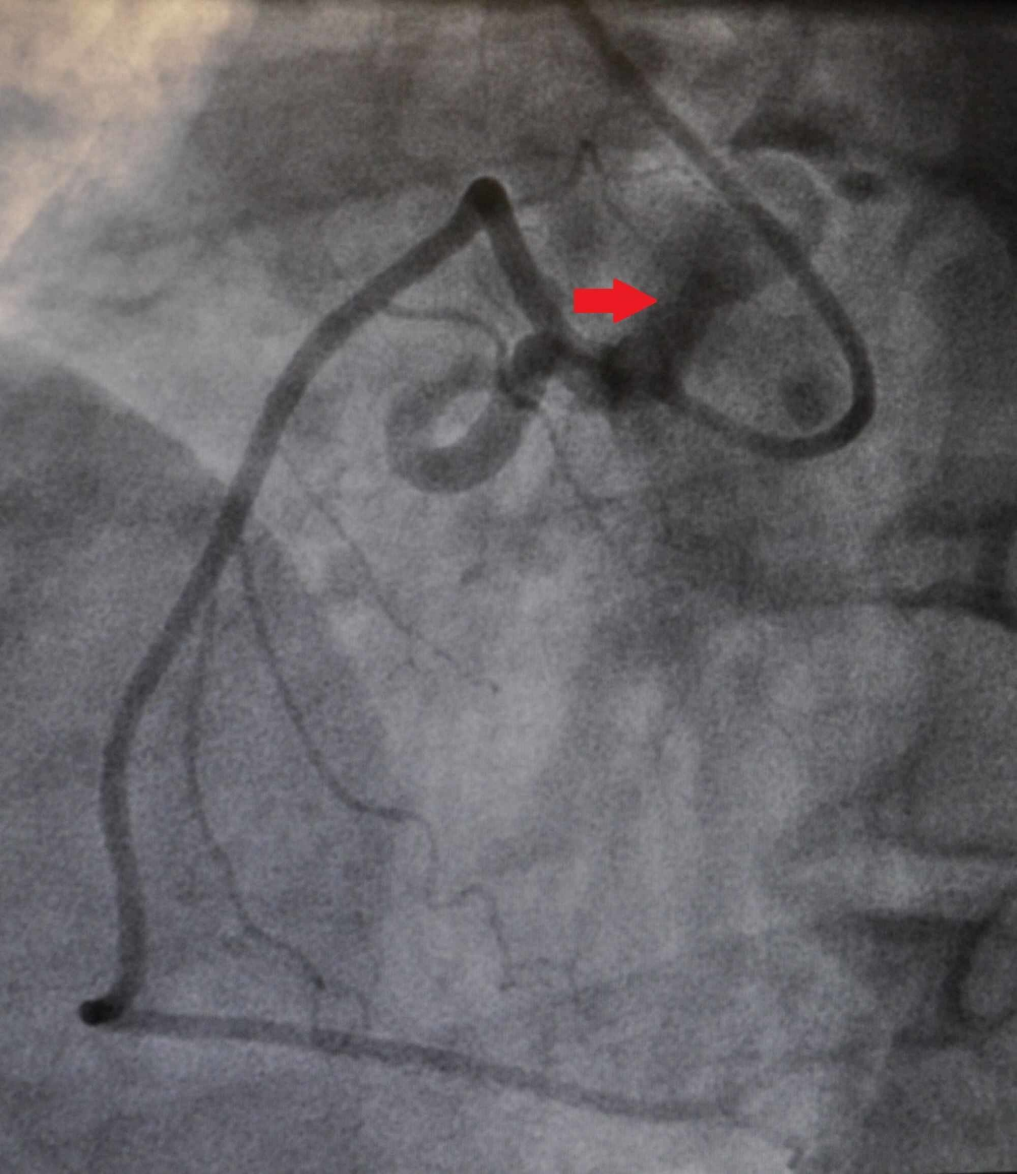
Right Coronary Artery to Pulmonary Artery Fistula (Red Arrow)

**Figure 3:**
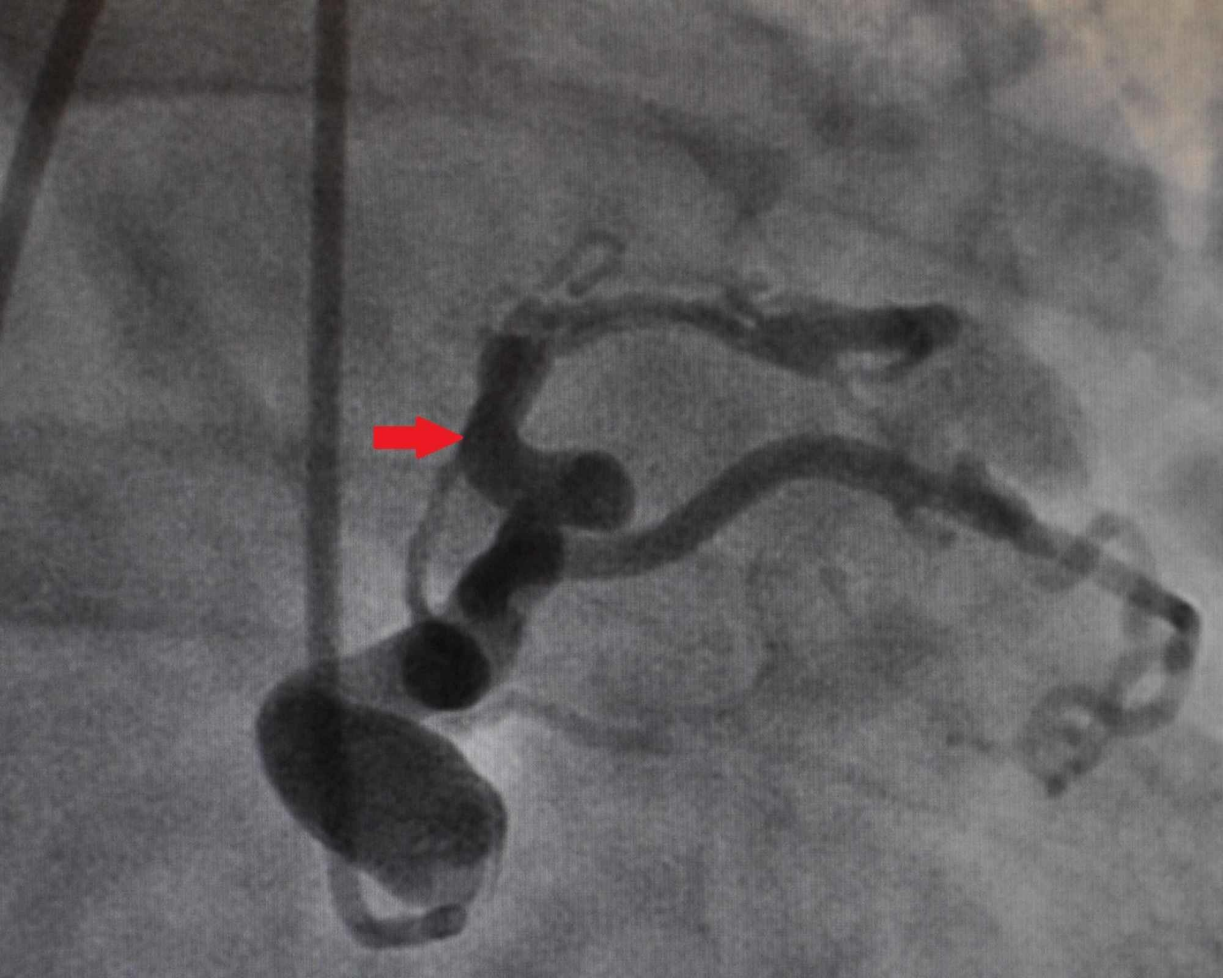
Aortic to Pulmonary Artery Fistula (Red Arrow)

A previously healthy 55-year-old male presented with complaints of progressive shortness of breath on exertion and fatigue. He underwent a stress test which showed anterolateral wall ischemia. This prompted a coronary angiogram revealing multiple tortuous CAFs, including a left coronary system to pulmonary artery (PA) fistula (Figure 1), a right coronary artery to PA fistula (Figure 2), and an aortic to PA fistula (Figure 3). He had no underlying coronary artery disease. Given his symptoms and significant left-to-right shunting with resultant coronary steal, the patient was taken to the operating room, in conjunction with cardiology, for ligation of the CAFs with the assistance of angiography.

A median sternotomy was made. The procedure was performed off-pump. Intraoperative coronary angiography was employed to aid in the identification of three of the fistulas. The origins and insertions of the CAFs were not entirely evident on gross examination as the courses they took were obscure and intraoperative angiography was crucial to the successful identification. Once clipped proximally and distally, the sharply transected, angiogram confirmed the complete ligation of each fistula. The patient had an uneventful hospital course, and he was discharged home on postoperative day 4.

## Discussion

CAFs are rare and usually single. When found they frequently originate from the right coronary artery system and connect with the right ventricle, right atrium, coronary sinus, or pulmonary arteries. Patients with CAFs can present with a variety of symptoms based on the patient’s age, amount of flow, and whether there is myocardial ischemia present [1]. In general, CAFs in the adult patient are asymptomatic, being found only on routine studies. Adult patients who are symptomatic present with dyspnea as their most common complaint. Demirkilic et al. examined 21 patients who underwent operative repair for CAFs with a mean age of approximately 37 years. Of these 21 patients, 29% complained of dyspnea with exertion [4]. Similarly, a study of 171 patients with CAFs by Rittenhouse et al. showed that when symptomatic, the most common complaint of patients was shortness of breath either while at rest or with exertion [5]. Our patient presented with progressive dyspnea with exertion, the most common symptom.

About half of the patients with large or multiple CAFs will develop complications, which include thrombosis, bacterial endocarditis, dissection, or rupture among others. Therefore, it is imperative to treat patients when they are symptomatic. As the first step to diagnosis of CAFs, coronary angiography is used and is considered the standard. This not only aids in demarcating the anatomy of the fistulas, but also helps with planning of closure [1,4]. The use of intraoperative angiography is less defined.

Although there is controversy surrounding the best treatment options for asymptomatic patients, closure of CAFs in symptomatic patients is recommended [6]. In fact, closure is necessitated in patients with considerable left-to-right shunt that in turn leads to ventricular overload [3]. Surgical options for CAFs include surgical ligation with or without coronary artery bypass grafting, and transcatheter closure [6]. In our patient with symptoms of congestive heart failure and tortuous CAFs, it was decided that surgical ligation would be the best option.

Transesophageal echocardiography (TEE) and intraoperative angiography, alone or in combination, have been reported to increase the successful closure rate of CAFs. There have been several reports documenting the utility of TEE. Takahashi et al. described a case of a 65-year-old male with an isolated CAF from the left anterior descending (LAD) artery to PA. Intraoperatively an abundance of vascular tissue was overlying the region of the fistula. TEE was used to assess the vascular flow to identify the neck of the fistula and its distal insertion for proper closure [7]. In another case report, Bouchez et al. described a 74-year-old female also with an LAD artery to PA fistula who underwent surgical ligation. The fistula closure was performed using TEE to help visualize the flow through the fistula and ensure its closure, noted by cessation of turbulent flow [8].

Less documented are surgical cases using intraoperative angiography as an adjunct to fistula closure. Kristian Hol et al. examined seven adult patients over the span of 10 years who had surgical intervention for CAFs. Four patients, who had their surgery in conjunction with image guidance, had sustained closure of their fistulas upon follow-up. In three of these four patients, intraoperative angiography identified more fistulas requiring ligation than originally thought present. These fistulas would not have been discovered otherwise. Two of the three patients, who underwent their surgery without the use of imaging, were noted to have left-to-right shunts at the time of follow-up [2].

There are no current recommendations for the utilization of intraoperative imaging in surgical CAF closure, and the option is left to the discretion of the operating surgeon. Given the complex anatomy and multiplicity of the CAFs encountered in our case, intraoperative coronary angiography was enlisted to first, correctly identify the origin and insertion of each fistula, and second, confirm definitive fistula closure. As each fistula had an elusive route, identification would not have been possible without the aid of on-table angiography. In sum, our case adds to the growing but limited body of literature supporting intraoperative imaging, in particular intraoperative angiography, for identification and successful ligation of CAFs.

## Conclusions

There is currently no consensus for using intraoperative coronary angiography in ligation of CAFs. Our case adds to the growing but limited body of literature supporting intraoperative imaging for identification and successful ligation of CAFs.

## References

[1] Luo L, Kebede S, Wu S, Stouffer GA (2006). Coronary artery fistulae. Am J Med Sci.

[2] Hol PK, Geiran O, Andersen K, Vatne K, Offstad J, Svennevig JL, Fosse E (2004). Improvement of coronary artery fistula surgery by intraoperative imaging. Ann Thorac Surg.

[3] Loukas M, Germain AS, Gabriel A, John A, Tubbs RS, Spicer D (2015). Coronary artery fistula: a review. Cardiovasc Pathol.

[4] Demirkilic U, Ozal E, Bingol H (2004). Surgical treatment of coronary artery fistulas: 15 years' experience. Asian Cardiovasc Thorac Ann.

[5] Rittenhouse EA, Doty DB, Ehrenhaft JL (1975). Congenital coronary artery—cardiac chamber fistula. Review of operative management. Ann Thorac Surg.

[6] Mangukia CV (2012). Coronary artery fistula. Ann Thorac Surg.

[7] Takahashi M, Wohler A, Abboud J, Sanz J, Kahn RA, Reddy RC (2012). Intraoperative imaging and off-pump ligation of coronary artery fistula. J Cardiothorac Vasc Anesth.

[8] Bouchez S, Coddens J, Vanermen H, Mustafa G, Shernan S (2001). Case 3—2001: multiplane transesophageal echocardiography in minimally invasive surgery for coronary artery fistula. J Cardiothorac Vasc Anesth.

